# Postmortem Serum Prostate-Specific Antigen as a Potential Marker for Prostatic Disease: A Forensic Exploratory Study

**DOI:** 10.7759/cureus.101818

**Published:** 2026-01-19

**Authors:** Sari Matsumoto, Shojiro Takasu

**Affiliations:** 1 Forensic Medicine, Jikei University School of Medicine, Minato City, JPN

**Keywords:** autopsy, benign prostatic hyperplasia, forensic pathology, postmortem biochemistry, prostate cancer, prostate-specific antigen, tumor markers

## Abstract

Introduction: Prostate-specific antigen (PSA) is widely used clinically to diagnose and monitor prostate cancer (PCa) and benign prostatic hyperplasia (BPH). Although PSA has long been used in forensic science for semen identification, its utility as a postmortem serum biomarker has not been systematically evaluated in a forensic autopsy context. This study explored whether postmortem serum PSA levels may reflect underlying prostatic pathology in forensic autopsy practice.

Methods: A total of 101 male autopsy cases (PCa, n = 3; BPH, n = 16; non-prostatic malignancies, n = 36; controls, n = 46) examined between 2015 and 2024 were included. Postmortem cardiac blood was collected, and serum PSA levels were measured using chemiluminescent enzyme immunoassays. Group comparisons were performed using the Kruskal-Wallis test with Dunn’s post-hoc analysis, and relationships between PSA levels, age, and postmortem interval (PMI) were examined using simple linear regression.

Results: Median PSA levels were 0.87 ng/mL in controls, 234.0 ng/mL in PCa, and 3.82 ng/mL in BPH, while PSA levels in non-prostatic malignancies were comparable to controls. Markedly elevated PSA values were observed in PCa cases compared with controls; however, all PCa-related findings were interpreted strictly as exploratory owing to the extremely small number of PCa cases (n = 3). In the BPH group, the PSA values showed only a modest tendency toward elevation relative to controls, with substantial overlap between groups. PSA levels in controls showed no significant correlation with age or PMI.

Conclusion: Postmortem serum PSA may serve as a supportive adjunctive indicator of underlying prostatic pathology in forensic autopsies; however, its diagnostic performance - particularly for distinguishing benign disease from normal aging - appears limited. These findings should be regarded as hypothesis-generating and require confirmation in larger multicenter cohorts. The primary objective was to explore whether postmortem serum PSA differs among PCa, BPH, and control cases, and the secondary objectives were to examine its relationships with age and PMI and the postmortem stability of PSA.

## Introduction

Prostate-specific antigen (PSA) is widely used in clinical medicine for the diagnosis and monitoring of prostate diseases. Elevated PSA levels occur in prostate cancer (PCa) and benign prostatic hyperplasia (BPH) [1-3]. Although PSA is mainly used in living patients, it may also provide useful postmortem information for identifying preexisting prostatic diseases. However, biochemical markers in postmortem samples are often affected by agonal changes and postmortem degradation, necessitating marker-specific evaluation of their stability and diagnostic relevance.

In forensic science, PSA has primarily been employed as a biomarker for semen identification in sexual assault investigations [4]. Studies have examined its stability under various conditions [5] and detectability even after decomposition [6]. Additionally, postmortem serum PSA levels have no clear correlation with age or the cause of death [7]. However, to the best of our knowledge, a systematic evaluation of postmortem serum PSA levels to distinguish PCa from BPH has not yet been reported. In forensic practice, occult prostatic disease is frequently encountered in elderly males, yet macroscopic identification of small or incidental lesions at autopsy can be challenging, highlighting the potential value of biochemical adjuncts such as postmortem PSA. Since advanced PCa can appear as poorly demarcated, grayish-white, hard lesions on autopsy, visual identification may be challenging, particularly when small cancers arise in the peripheral zone and are confused with nodular hyperplasia. Therefore, assessing whether postmortem PSA measurements can assist in identifying prostatic diseases has considerable forensic value.

To our knowledge, this is the first study to systematically evaluate postmortem serum PSA as a biochemical adjunct for identifying prostatic diseases across malignant, benign, and control groups in forensic autopsy practice.

In this study, we compared postmortem serum PSA levels among PCa, BPH, non-prostatic malignancies, and control cases. PSA values were evaluated descriptively and in relation to conventional clinical thresholds. Additionally, we examined the influence of age and postmortem interval (PMI) on PSA levels. Accordingly, this study was designed as an exploratory investigation with the primary objective of assessing differences in postmortem serum PSA among PCa, BPH, and control cases, and secondary objectives of examining potential confounding effects of age and PMI, as well as the postmortem stability of PSA.

## Materials and methods

Case selection

Between July 1, 2015, and March 31, 2024, a total of 101 male autopsy cases were examined at our institution in Japan. Only cases with at least 0.5 mL of postmortem serum available for PSA measurement were included in the analysis. Antemortem clinical diagnoses were reviewed but were not used as primary criteria for case selection or group assignment.

The participants were grouped as follows: the PCa group (n = 3), consisting of cases in which PCa was confirmed by macroscopic autopsy findings with histopathological confirmation; the BPH group (n = 16), comprising cases diagnosed with BPH based on autopsy findings with histopathological confirmation; and the control group (n = 46), which included cases with no malignant tumors, prostate disease, or documented clinical history of prostate disorders. All PCa and BPH diagnoses were established using a combination of macroscopic autopsy findings and histopathological confirmation, with microscopic examination regarded as the diagnostic gold standard in all cases.

The causes of death in the control group included acute myocardial infarction (n = 11), subarachnoid hemorrhage (n = 2), cerebral hemorrhage (n = 7), pneumonia (n = 11), drowning (n = 6), asphyxia (n = 6), and intoxication (n = 3). These three groups comprised the main cohort.

Additionally, a supplementary analysis was conducted for the non-prostatic malignancy group (n = 36), which included cases with malignant tumors other than PCa: malignant lymphoma (n = 3), esophageal cancer (n = 4), gastric cancer (n = 7), colorectal cancer (n = 2), rectal cancer (n = 6), hepatocellular carcinoma (n = 3), cholangiocarcinoma (n = 3), and lung cancer (n = 7).

Cases with a PMI exceeding 120 hours, female cases, or cases with a history of treatment for prostatic disease were excluded. When overlapping conditions were present, classification was prioritized based on the presence of PCa.

Sample collection and measurement

Cardiac blood was collected during autopsy, centrifuged at 3,000 rpm for 10 minutes, and the obtained serum was stored at -80 °C until analysis. Samples were visually inspected for gross hemolysis; however, no formal grading or quantitative assessment of hemolysis was performed. The duration of frozen storage before analysis varied among cases and was not systematically recorded.

Serum PSA concentrations were measured once for each case, but the analytical laboratory differed depending on the period of examination. Specifically, a portion of the samples was analyzed by SRL, Inc. (Tokyo, Japan), using the Lumipulse Presto® PSA assay manufactured by Fujifilm Wako Pure Chemical Corporation (Osaka, Japan), whereas the remaining samples were analyzed by LSI Medience Corporation (Tokyo, Japan) using the ARCHITECT® PSA platform produced by Abbott Laboratories (Abbott Park, IL). Both laboratories used standardized chemiluminescent enzyme immunoassay (CLIA) methods with validated quality-control procedures. The detection limit was 0.4 ng/mL, and the clinical reference value was 4.0 ng/mL [8]. No formal cross-calibration between the two assay platforms was performed.

Histopathological examination

Representative tissue sections from the prostate and other organs were prepared and stained with hematoxylin and eosin. Histological diagnoses were performed by forensic pathologists with over 10 years of experience, based on the World Health Organization Classification of Tumours, 5th Edition.

Statistical analysis

In the control group, correlations among PSA level, age, and PMI were examined using simple linear regression. Group comparisons of PSA concentrations among the study groups were performed using the Kruskal-Wallis test followed by Dunn's multiple comparison test. Proportions exceeding the conventional clinical cutoff value of 4.0 ng/mL were compared using Fisher's exact test.

Receiver operating characteristic (ROC) curves were constructed to evaluate the diagnostic performance for BPH and for the combined prostatic disease group. Optimal cutoff values were determined using the Youden index, and sensitivity, specificity, and area under the curve (AUC) were calculated for these groups. PCa-related ROC analyses were performed for exploratory purposes only and are not presented because of the extremely small number of PCa cases.

All statistical analyses were performed using GraphPad Prism (version 10.0; GraphPad Software, San Diego, CA) and Microsoft Excel (Microsoft Corp., Redmond, WA). Statistical significance was set at p < 0.05.

Ethical approval

This study was approved by the Ethics Committee of Jikei University School of Medicine, Tokyo, Japan (Approval No. 34-160(11311)).

## Results

A total of 101 male autopsy cases met the inclusion and exclusion criteria described in the Methods section and were included in the final analysis. Based on macroscopic autopsy findings with histopathological confirmation, these cases were classified into four groups: prostate cancer (PCa, n = 3), benign prostatic hyperplasia (BPH, n = 16), controls without prostatic disease (n = 46), and a supplementary non-prostatic malignancy group (n = 36). This distribution reflects the final study population analyzed in subsequent comparisons.

The baseline characteristics of the 101 male autopsy cases are summarized in Table 1. The BPH group was significantly older than the control group (p < 0.0001). No significant differences in PMI were observed among the groups.

**Table 1:** Baseline characteristics of the study population † Kruskal–Wallis test for age among groups (H=25.93, p<0.0001). ‡ Kruskal–Wallis test for PSA among groups (H=26.21, p<0.0001). Abbreviations: AMI, acute myocardial infarction; ICH, intracerebral hemorrhage; SAH, subarachnoid hemorrhage; PMI, postmortem interval; PSA, prostate-specific antigen; BPH, benign prostatic hyperplasia; PCa, prostate cancer

Variables	Control	PCa	BPH	Non-PCa malignancies	p value
Age, years, median (IQR)	60.50 (49.75–72.00)	79.50 (77.00–90.00)	80.00 (74.00–87.50)	66.50 (53.00–75.50)	<0.0001†
PMI, hours, median (IQR)	41.00 (33.75–53.00)	39.00 (17.00–79.00)	33.50 (24.25–68.25)	43.00 (31.25–53.75)	0.79
PSA concentration, ng/ml, median (IQR)	0.87 (0.45–3.27)	234.0 (133.0–958.0)	3.82 (1.96–26.53)	4.94 (0.40–5.85)	<0.0001‡
Cause of death, n(%) (control group)					
AMI	11 (23.9)	-	-	-	
Cerebrovascular (ICH, SAH)	9 (19.6)	-	-	-	
Pneumonia	11 (23.9)	-	-	-	
Asphyxia/drowning	12 (26.1)	-	-	-	
Poisoning	3 (6.5)	-	-	-	
Malignant diseases type (non-PCa malignancies group)					
Lymphoma	-	-	-	3 (8.3)	
Esophageal cancer	-	-	-	4 (11.1)	
Gastric cancer	-	-	-	7 (19.4)	
Colorectal cancer	-	-	-	8 (22.2)	
Liver/bile duct cancer	-	-	-	6 (16.7)	
Lung cancer	-	-	-	7 (19.4)	

**Figure 1:**
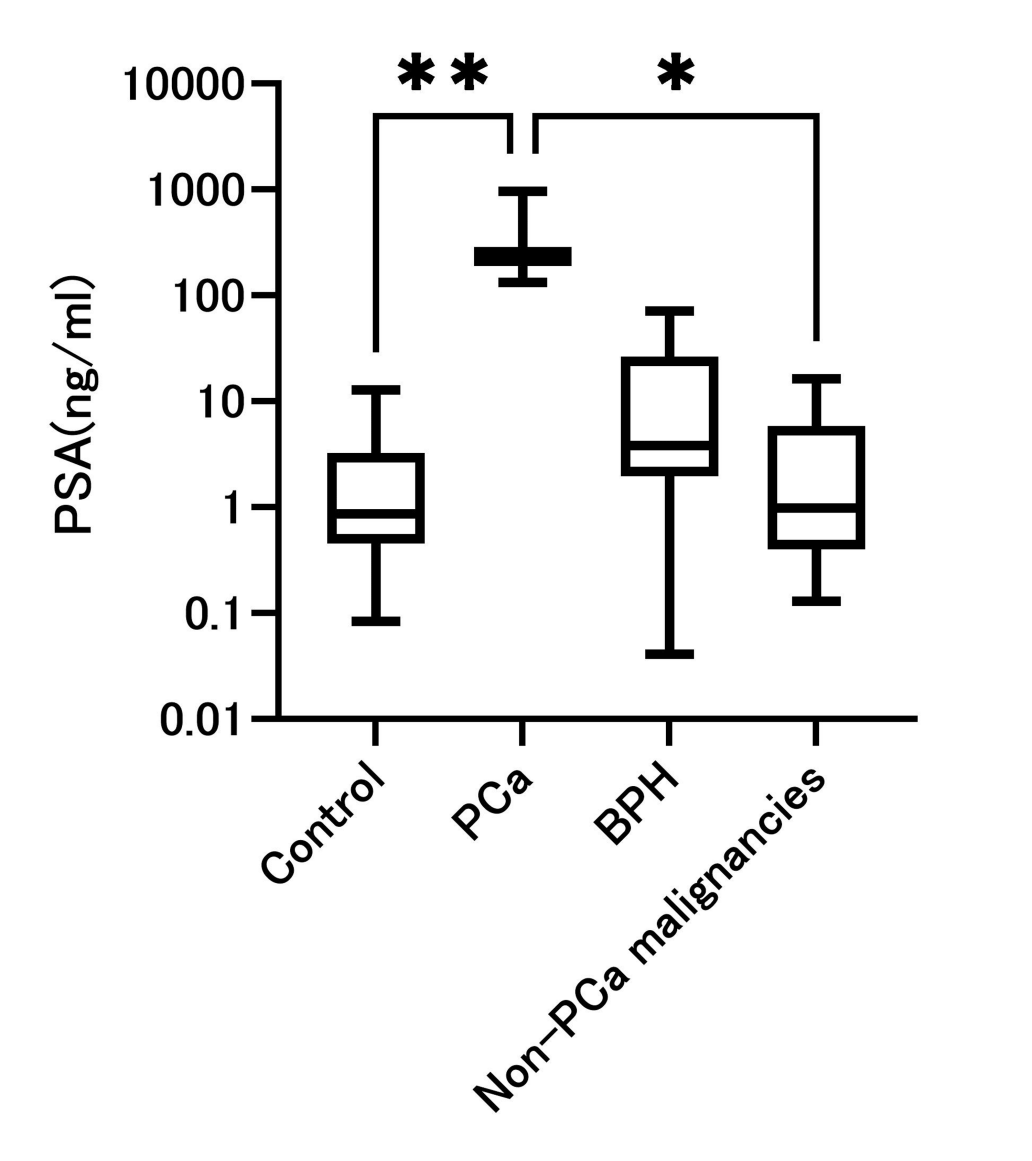
Distribution of postmortem serum PSA levels among the study groups Box-and-whisker plots showing postmortem serum prostate-specific antigen (PSA) concentrations in the control group, prostate cancer (PCa) group, benign prostatic hyperplasia (BPH) group, and the combined “prostate disease” group (PCa + BPH). Boxes represent the interquartile range (IQR), horizontal lines indicate the median values, and whiskers denote the minimum and maximum observations within 1.5 × IQR. The PSA values were plotted on a log10 scale to account for a wide dynamic range. Abbreviations: PSA, prostate-specific antigen; PCa, prostate cancer; BPH, benign prostatic hyperplasia.

The PCa group showed markedly higher PSA levels than the control group (p = 0.0088); however, this finding should be interpreted cautiously because only three PCa cases were available (Table 1, Figure 1). The BPH group showed a trend toward higher PSA levels than the control group. However, this difference was not statistically significant (p = 0.065). When PCa and BPH were combined as the “prostatic disease group,” PSA levels were significantly higher than in controls (p = 0.0034). No significant differences were observed between the PCa and BPH groups (p = 0.5791). In the supplementary analysis, the non-prostatic malignancy group showed PSA values comparable to those of the control group (p > 0.99).

**Figure 2:**
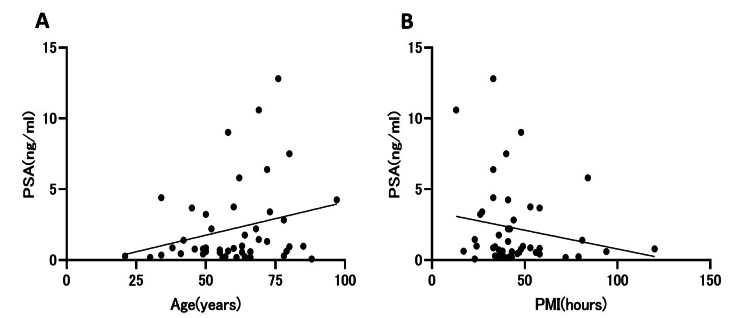
Scatter plots of postmortem serum PSA levels in the control group (A) Relationship between PSA and age (simple linear regression; no significant correlation, p = 0.083, R² = 0.067). (B) Relationship between PSA and PMI (p = 0.22, R² = 0.034). Regression lines are shown for visualization. Abbreviations: PSA, prostate-specific antigen; PMI, postmortem interval

In the control group, simple linear regression analysis revealed no significant association between age and serum PSA levels. The regression model demonstrated a p-value of 0.083 and an R² of 0.067, indicating that age did not meaningfully influence PSA concentrations within the early postmortem period (Figure 2). Similarly, serum PSA levels showed no significant relationship with PMI in the control group, with a p-value of 0.22 and an R² of 0.034.

Using the conventional clinical cutoff of 4.0 ng/mL, binarized analysis showed that 7/16 BPH cases (43.8%) and 8/46 control cases (17.4%) exceeded the cutoff, indicating substantial overlap between the two groups. The proportion exceeding the cutoff was significantly higher in the BPH group than in the control group (p = 0.046). When PCa and BPH were combined into a broader prostatic disease group, the proportion exceeding the cutoff was also significantly higher than in controls (p = 0.0063). Because only three PCa cases were available, PCa-specific diagnostic performance metrics were not calculated or presented.

**Figure 3:**
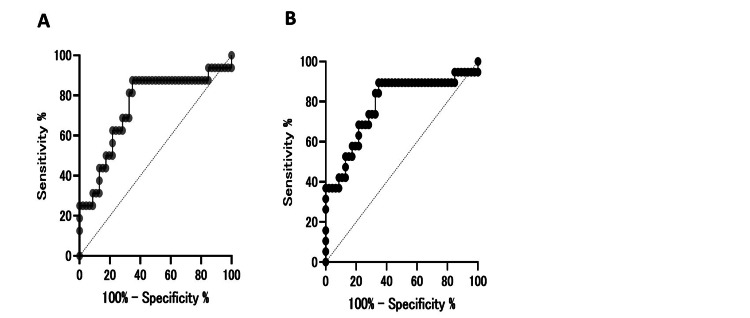
Receiver operating characteristic curves for PSA in distinguishing prostate disease from controls (A) ROC curve for benign prostatic hyperplasia (BPH) versus control group (AUC = 0.74; optimal cutoff 1.61 ng/mL). (B) ROC curve for the combined prostatic disease group (exploratory analysis). The diagonal dashed line indicates a line with no discrimination (AUC = 0.5). The optimal cutoff values were determined using the Youden index. PSA, prostate-specific antigen; AUC, area under the curve.

ROC curve analysis for BPH yielded an AUC of 0.74, with an optimal cutoff value of 1.61 ng/mL determined using the Youden index (Table 2, Figure 3). For the combined prostatic disease group, the AUC was 0.78 with the same exploratory cutoff value. In the PCa group, PSA values were markedly higher than in controls, showing apparent separation in this limited dataset; however, formal ROC-derived diagnostic performance metrics and cutoff values were not presented because of the extremely small number of PCa cases. These ROC-based analyses were performed solely for exploratory purposes and should not be interpreted as validation of diagnostic thresholds.

**Table 2:** Diagnostic performance of postmortem serum PSA using conventional and ROC-derived cutoff values PCa-related diagnostic performance metrics were intentionally omitted because only three PCa cases were available, and any ROC-derived estimates were considered unreliable and unsuitable for diagnostic interpretation. * Fisher exact test was performed.

Group	Cutoff (ng/mL)	Sensitivity % (95% CI)	Specificity % (95% CI)	PPV % (95% CI)	NPV % (95% CI)	p value*
BPH vs. control	4	43.75 (23.10–66.85)	82.61 (69.28–90.91)	46.67 (24.81–69.88)	80.85 (67.46–89.58)	0.046
	1.61 (ROC-derived)	87.50 (63.98–97.78)	34.78 (22.68–49.23)	31.82 (20.00–46.56)	88.89 (67.20–98.03)	0.12
Prostate disease† vs. control	4	52.63 (31.71–72.67)	82.61 (69.28-90.91)	55.56 (33.72–75.44)	80.85 (67.46–89.58)	0.0063
	1.61 (ROC-derived)	89.47 (68.61–98.13)	34.78 (22.68–49.23)	36.17 (23.97–50.46)	88.89 (67.20–98.03)	0.068

## Discussion

This study explored postmortem serum PSA levels in male autopsy cases to assess whether PSA may reflect underlying prostatic pathology in a forensic setting. Markedly elevated PSA values were observed in PCa cases compared with controls; however, formal diagnostic performance metrics and cutoff values are not presented for PCa because only three cases were available, and the apparent separation observed in this limited dataset is most likely an artifact of complete separation. In the BPH group, PSA values showed only a modest tendency toward elevation relative to controls, with substantial overlap between groups, indicating limited discriminatory ability. When PCa and BPH were combined into a broader “prostatic disease” category, PSA levels remained significantly higher than in controls, suggesting that postmortem PSA may serve as a general adjunctive indicator of prostatic pathology rather than a disease-specific diagnostic marker. This combined analysis was performed solely to increase statistical power in this small exploratory cohort and should not be interpreted as implying biological equivalence between malignant and benign prostatic disease. All findings should therefore be interpreted strictly as exploratory and hypothesis-generating. To our knowledge, this is the first study to systematically examine postmortem serum PSA across malignant, benign, and control groups in a forensic autopsy cohort.

Utility of PSA in prostatic disease

PSA is an androgen-regulated serine protease belonging to the kallikrein family and is widely used for the prediction, detection, and monitoring of prostate cancer, as well as for assessing prostatic enlargement [1,9,10]. Its biological characteristics, including complex formation with protease inhibitors and glycosylation, contribute to its relative stability in circulation [10]. However, PSA is not tumor-specific; its diagnostic accuracy is substantially influenced by benign prostatic enlargement and inflammatory conditions, resulting in false-positive findings and potential overdiagnosis in clinical screening [7,8,11-14].

The significantly elevated postmortem PSA levels observed in PCa cases in this study are consistent with established clinical findings [1,7]. However, the apparent separation of PSA values observed in this limited PCa dataset most likely reflects complete separation due to the extremely small number of cases rather than true diagnostic perfection. As emphasized in large screening cohorts, clinically meaningful PCa can occur even at PSA values ≤4.0 ng/mL [7], indicating that postmortem cutoff values must be interpreted with caution and cannot be directly extrapolated from clinical practice. Larger studies are therefore required to define valid postmortem reference ranges and thresholds.

In the BPH group, PSA elevation was frequently observed, but substantial overlap with controls limited diagnostic specificity. This mirrors clinical observations in which benign prostatic enlargement contributes significantly to PSA elevation and limits discrimination between malignant and benign disease [8,11]. Accordingly, elevated postmortem PSA should not be interpreted as indicative of malignancy in isolation, and BPH must be considered in the differential diagnosis when gross or histological findings are inconclusive. Although a lower cutoff improved sensitivity for detecting BPH, this occurred at the expense of markedly reduced specificity. These findings indicate that postmortem PSA is poorly suited for distinguishing BPH from normal aging and that its forensic value lies primarily in excluding significant prostatic pathology rather than confirming benign disease. The PCa and BPH groups were significantly older than the control group, and age is a well-established determinant of both PSA levels and the prevalence of occult prostatic disease. Although no correlation between age and PSA was observed within the control group, this does not adequately address confounding by age across diagnostic categories. Because only three PCa cases were available, multivariable modeling adjusting for age and other confounders was statistically inappropriate and therefore not attempted. The absence of age-adjusted analyses should thus be regarded as a key limitation when interpreting the observed group differences.

Specificity of PSA and future biomarker perspectives

The non-PCa malignancy group demonstrated PSA values comparable to those of the controls, reinforcing the organ specificity of PSA even in postmortem serum. This finding suggests that PSA elevation is predominantly associated with prostatic pathology rather than with generalized malignancy. Historically, PSA has primarily been used for semen identification in forensic applications [4]. The present study expands its forensic utility by demonstrating that serum PSA levels can aid in identifying occult or incidental prostatic diseases during autopsy.

Recent reviews have emphasized that PSA alone is insufficient as a cancer-specific biomarker because its diagnostic accuracy is compromised by benign prostatic conditions, inflammatory states, and age-related changes [7,8,11,12,15]. Bratt et al. summarized that, although PSA remains the most widely used serum marker, its sensitivity and specificity are suboptimal, leading to false-positive results and overdiagnosis in clinical screening [15]. Furthermore, systematic reviews have highlighted the growing interest in alternative blood- and urine-based biomarkers, including kallikrein panels, PCA3, and TMPRSS2-ERG, which may improve diagnostic precision when combined with PSA [16]. Prensner et al. proposed that multimarker strategies integrating molecular biomarkers with conventional PSA testing represent the next step in prostate cancer detection [17]. From a forensic perspective, such integrated biomarker approaches may eventually enable more accurate postmortem discrimination between malignant and benign prostatic diseases, particularly in cases in which gross or histological findings are inconclusive.

Postmortem stability and technical considerations

Although postmortem biochemical markers are often affected by autolysis and proteolytic degradation, PSA appears to retain relative stability during the early postmortem period. In this study, no significant correlations were observed between PSA levels, age, or PMI among the controls, suggesting that PSA remained relatively stable during the early postmortem period (within approximately 120 hours). These findings are consistent with previous reports showing that PSA retains its detectability in biological fluids under postmortem conditions [5]. Tsuboi et al. similarly reported that serum PSA levels in cadavers correlate with age in a manner comparable to living subjects, suggesting preservation of biologically meaningful PSA information after death [6]. Moreover, in vitro studies have demonstrated that total PSA remains relatively stable under controlled storage conditions [18], supporting the feasibility of postmortem PSA measurement. Nevertheless, the influence of prolonged PMI, advanced decomposition, and sampling site (e.g., cardiac vs peripheral blood) remains to be clarified.

Forensic implications

Unlike clinical screening, postmortem PSA measurement is not influenced by pre-analytical factors, such as digital rectal examination, ejaculation, or acute inflammatory states, suggesting that postmortem PSA may, in some circumstances, reflect intrinsic prostatic pathology with fewer pre-analytical confounders than antemortem values. In elderly males, in whom occult PCa and BPH are frequently encountered, macroscopic identification of small peripheral-zone carcinomas can be challenging [2]. In such settings, serum PSA may provide valuable supplementary information to guide focused histopathological examination and enhance the interpretation of pre-existing disease in forensic investigations.

Limitations

These limitations substantially constrain the inferential strength of the present findings and require that all results be interpreted strictly as hypothesis-generating.

The present study has several important limitations. First, the number of PCa cases was extremely small (n = 3), which precluded reliable estimation of diagnostic performance metrics and rendered ROC-derived cutoff values highly unstable. For this reason, specific ROC-based performance measures for PCa were not presented, and all PCa-related analyses were interpreted strictly as exploratory. The PCa and BPH groups were significantly older than controls, and the absence of age-adjusted multivariable analyses should be considered a limitation of this exploratory study. Second, this was a single-center retrospective study, which may restrict the generalizability of the findings. Third, only cases with a postmortem interval of ≤120 hours were included; therefore, the behavior and stability of PSA in cases with longer PMI or advanced decomposition remain unknown. Fourth, pre-analytical factors specific to postmortem biochemistry - including potential differences between cardiac and peripheral blood sampling sites, redistribution effects, hemolysis, and postmortem diffusion - were not systematically evaluated and may have influenced measured PSA concentrations. Finally, serum PSA was measured using two different immunoassay platforms without formal cross-calibration or validation studies. Therefore, minor inter-assay variability cannot be excluded and may have influenced PSA concentrations, particularly for values near diagnostic cutoffs.

## Conclusions

This study demonstrated that postmortem serum PSA levels can serve as a useful adjunctive indicator of underlying prostatic pathology, particularly prostate cancer, in forensic autopsy practice. PSA concentrations were markedly elevated in PCa cases and modestly increased in BPH cases, whereas levels in non-prostatic malignancies remained comparable to controls, highlighting the organ-specific nature of PSA even after death. These findings support the potential forensic relevance of PSA measurement when macroscopic or histopathological findings are subtle or inconclusive. Larger multicenter studies are warranted to further validate these observations and to establish standardized postmortem reference values.
